# Integrated care in France: dream or reality?

**DOI:** 10.5334/ijic.1540

**Published:** 2014-03-27

**Authors:** Dominique Somme

**Affiliations:** Faculté de Médecine, Université de Rennes 1, France

The French Society of Geriatrics and Gerontology has published a position paper in French on its view of the concept of integrated care and how it dovetails with the concept of coordination. Now, the society is grateful to the *International Journal of Integrated Care* for this opportunity to explain its stance to an international English-language readership [1].

Our participation at meetings of the International Conference of Integrated Care and our involvement with the International Foundation for Integrated Care show that our French experience can be instructive for health and social care professionals, including geriatricians, in other countries, and also for decision-makers, managers, directors and all gerontologists interested in the complex concept of ‘integration’ and how it relates to the concept of ‘coordination between organisations’.

However, the today publication of this article may also seem somewhat surprising to those people most familiar with the current organisation of the French system of health and social care and its capacity for reform.

When the Society of Geriatrics and Gerontology working group on integrated care was set up, integration was part of public policy in France, as reported in the *International Journal of Integrated Care* [2]. Through the creation of Homes for the Integration and Autonomy of Alzheimer patients, or ‘MAIAs’, an attempt was made to adapt the concept of integration in its entirety to the multifaceted reality that is France. This policy was based on a study, Project and Researches on Integration for the Maintenance of Autonomy in France (PRISMA France), which grew out of a unique and original adaptation of a model from Quebec to the very different reality of the French system [3–5]. This policy put France in a position of innovation, backed by a research team, something unusual in our country in public policy management.

Alas, two years later it is now obvious that integration has completely disappeared from political debate and that this public policy (that of the MAIAs), although ongoing, is gradually being stripped of its relation to integration as defined in the article that we are publishing today. Yet, everything that has been published in French, and in English, points to real and observable changes on the ground (albeit often still small) and to France's capacity to move towards more integrated care by strengthening this policy [2].

Since the 2012 change of government in France, none of the important texts emanating from or submitted to the relevant ministries with a view to drafting future laws cite the research work conducted hitherto. When the MAIAs are mentioned it is often as if they were a mechanism of coordination, or simply that the idea was to put in place case management. The fact that integration is not at the heart of these policies in no way, of course, diminishes their other virtues (notably, giving pride of place to prevention is unquestionably crucial), but it is a pity that so much effort by the public authorities has been overlooked.

## How can we explain what has led to this apparent paradox?

**Figure 1.**
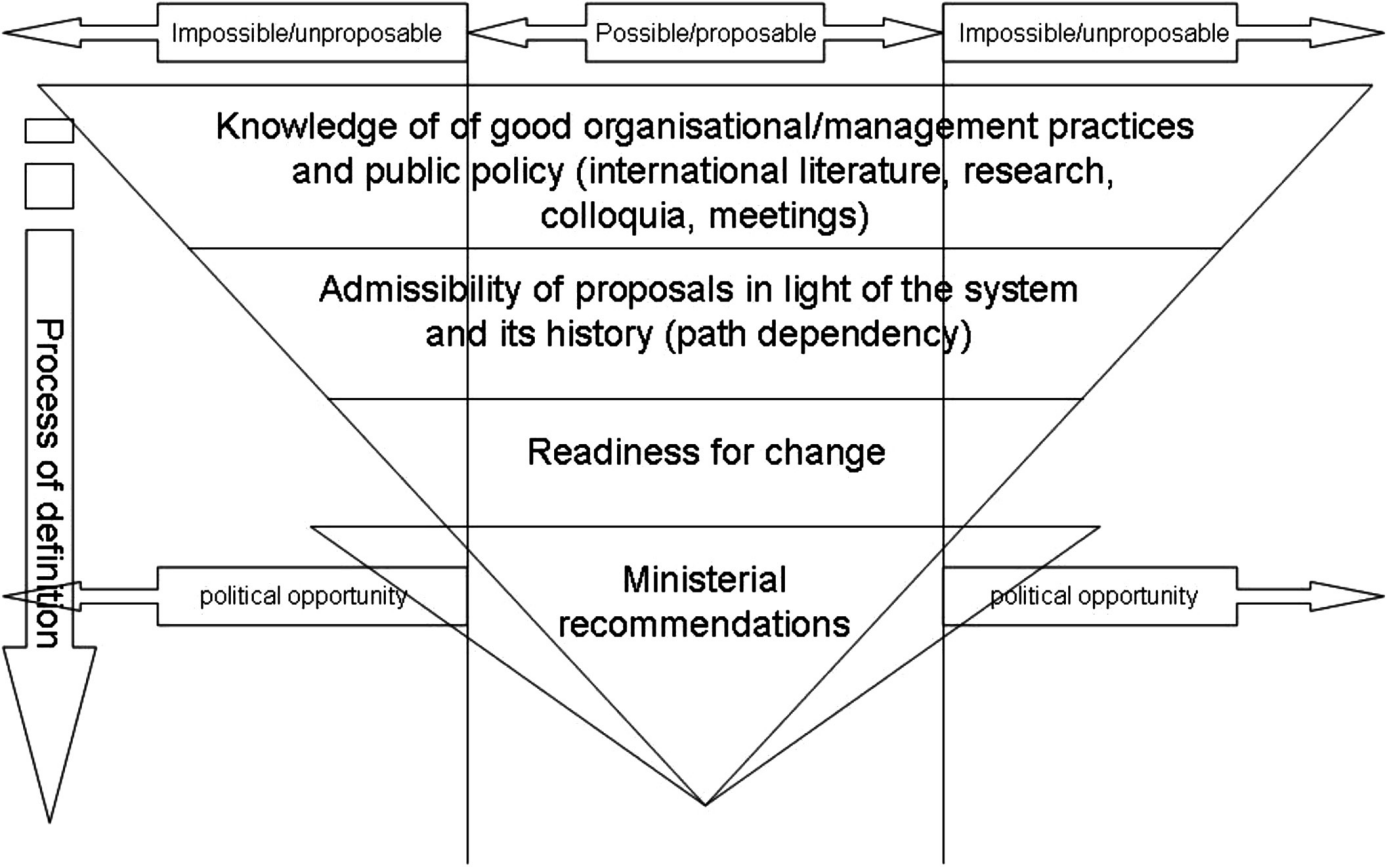
Definition of a policy.

It seems that all public policies stem from a process operating on two time scales. The first stage, outlined in Figure 1, is that of the definition of ministerial guidance, generally through adaptation of know-how to historical possibilities [6] or to opportunities for change [7].

**Figure 2.**
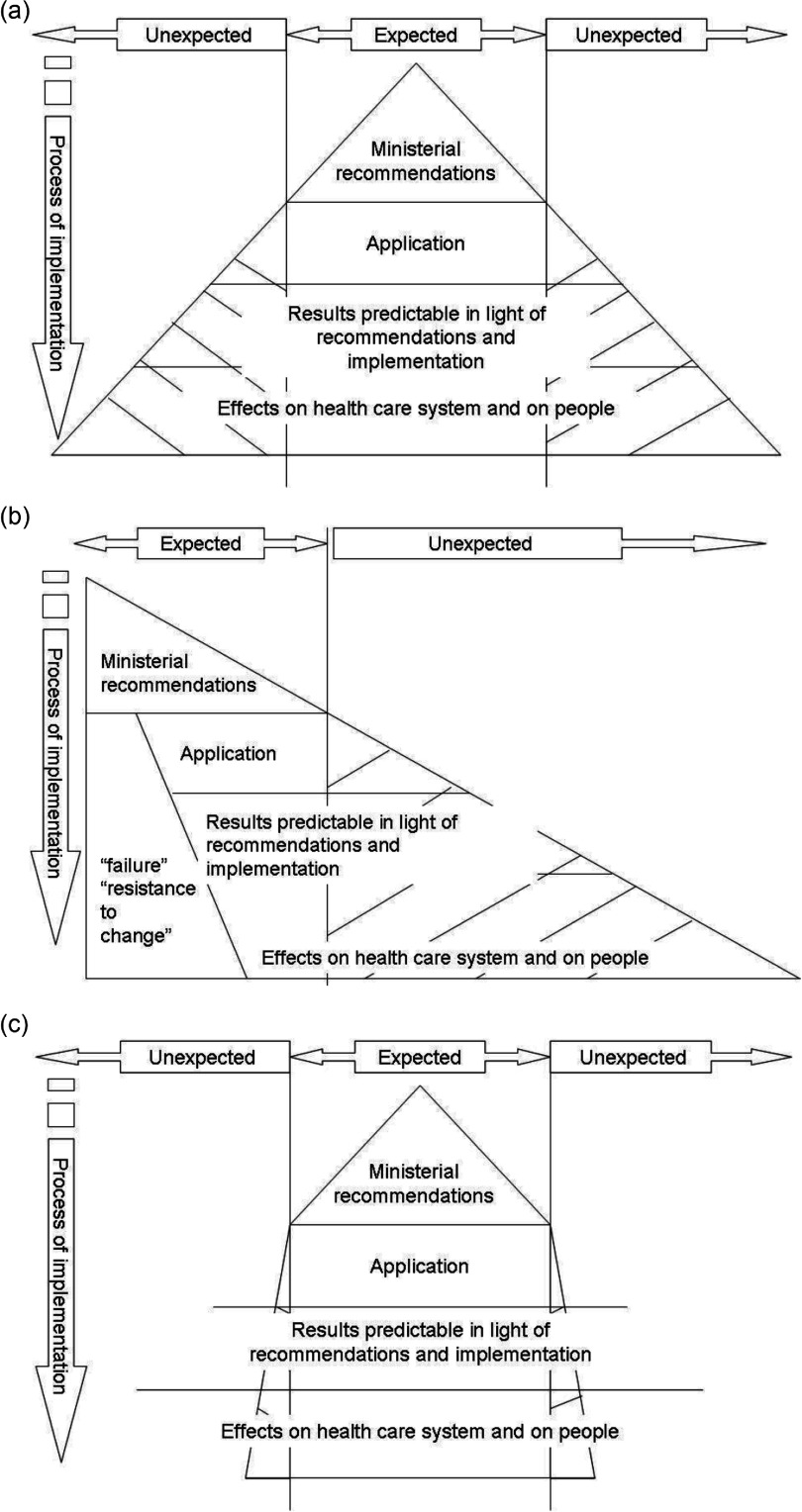
Implementation of a programme as a function of the intervention.

The second stage, shown in Figure 2, is the progression from guidance to implementation. In France, we have documented a certain acceptance of and readiness for change. There is, however, the possibility of a policy shift related to political opportunities, which also underpin original policies. Two major shifts were introduced at the time of the implementation of the proposals suited to the French system: a link with a policy dedicated to Alzheimer's and related diseases and the use of the name ‘homes’ in designating the innovation. It has already been documented that integrated care is not defined disease by disease (see the position statement article) and the term ‘home’ suggests the existence of a new structure rather than a new method of organising the existing structure (which was, in fact, proposed) [2].

In applying a policy, there is always a measure of the unexpected. Figure 2 presents possible implementations taking into account the type of intervention.

Figure 2a corresponds to a ‘let-it happen’ intervention [8]. Of course we know the content of the recommendations, but their implementation deviates a little at the outset, leaving aside certain aspects and often developing innovative ‘compensatory’ mechanisms. The results expected at the planning decision stage are often incompletely achieved and unforeseen results may emerge. So the effects on the system are by nature unpredictable and can in fact increase its complexity. Figure 2b shows a ‘make-it happen’ intervention [8]. Here, at the other end of the governance spectrum, the whole plan will be implemented with few innovations, and without adaptation to local realities. The impact on the system will be modest (notably because of the smallness of the ‘unexpected’ impact). This presupposes a virtually unique chain of decision from the highest level to the end-users. This appears unsuited to French health and social care systems characterised by a highly complex governance of services. Figure 2c illustrates a ‘help-it happen’ intervention [8]. Here, the bulk of the work will be to ensure that the implementation is consistent with the basic features of the plan, notably the aims of transformation. Negotiation and knowledge transfer must be rooted in a clear statement of intent from all those who manage support systems, and policy decisions must have a genuine impact.

After the change of government in France in 2012, we witnessed a rather sudden shift from a situation close to that of Figure 2c during the ‘experimental phase’ of the plan (dedicated project team linked directly to executive power) to a situation close to that of Figure 2a (far fewer direct ties with the political system and development of concurrent policy without dialogue with those responsible for the experimental phase).

## So, is an assessment possible? Where does integrated care now stand in France?

The picture is a mixed one. There is an ongoing policy, but its political focus is insufficient to achieve the aims of in-depth transformation of the system and to press home the message on integrated care. Furthermore, monitoring of the process through research has been curtailed, which may blur the real implementation of the model. All that exists and that has been implemented is waiting for someone to ‘talk’ about integrated care. An absolute rule of integrated care, as the reader is doubtless well aware, is that it does not emerge naturally, because it is in tension with many different interests. With tensions between ‘the natural slope’ and ‘the choice of a promising but risky path’, what makes the difference is political discourse. In, for example, Singapore (which has a National Agency for Integrated Care), Quebec, the Netherlands and the United Kingdom, integrated care is seen as a solution to problems identified as priorities for health and social care systems.

Like other countries, France will in the end be ‘pushed’ towards integrated care by the epidemiological, demographic and economic challenges we face. And by the influence people wield over organisation of the health and social care system. If we wish to have ‘activated’ users of the health care system, as suggested by the chronic care model [9], users must understand the system. The final challenge is to focus on where people live, to make the transition from a hospital-based system to a home-based system.

In conclusion, one may wonder whether French society is ready for this change. In a recent regional referendum on the simplification of political institutions, not enough people voted for the result to be valid. A satirical newspaper subsequently ran a piece entitled ‘Long Live the Mille-feuille!’, in an allusion to what might be deemed a national attachment to multilayered complexity.
